# Cellular Uptake of Psychostimulants – Are High- and Low-Affinity Organic Cation Transporters Drug Traffickers?

**DOI:** 10.3389/fphar.2020.609811

**Published:** 2021-01-20

**Authors:** Ole Jensen, Muhammad Rafehi, Lukas Gebauer, Jürgen Brockmöller

**Affiliations:** Institute of Clinical Pharmacology, University Medical Center Göttingen, Göttingen, Germany

**Keywords:** membrane transport, monoamine transporter, OCT1, organic cation transporter, psychostimulant, SLC22A1, solute carrier, hallucinogen

## Abstract

Psychostimulants are used therapeutically and for illegal recreational purposes. Many of these are inhibitors of the presynaptic noradrenaline, dopamine, and serotonin transporters (NET, DAT, and SERT). According to their physicochemical properties, some might also be substrates of polyspecific organic cation transporters (OCTs) that mediate uptake in liver and kidneys for metabolism and excretion. OCT1 is genetically highly polymorphic, with strong effects on transporter activity and expression. To study potential interindividual differences in their pharmacokinetics, 18 psychostimulants and hallucinogens were assessed *in vitro* for transport by different OCTs as well as by the high-affinity monoamine transporters NET, DAT, and SERT. The hallucinogenic natural compound mescaline was found to be strongly transported by wild-type OCT1 with a *K*_*m*_ of 24.3 µM and a *v*_max_ of 642 pmol × mg protein^−1^ × min^−1^. Transport was modestly reduced in variants *2 and *7, more strongly reduced in *3 and *4, and lowest in *5 and *6, while *8 showed a moderately increased transport capacity. The other phenylethylamine derivatives methamphetamine, *para*-methoxymethamphetamine, (-)-ephedrine, and cathine ((+)-norpseudoephedrine), as well as dimethyltryptamine, were substrates of OCT2 with *K*_*m*_ values in the range of 7.9–46.0 µM and *v*_max_ values between 70.7 and 570 pmol × mg protein^−1^ × min^−1^. Affinities were similar or modestly reduced and the transport capacities were reduced down to half in the naturally occurring variant A270S. Cathine was found to be a substrate for NET and DAT, with the K_m_ being 21-fold and the *v*_max_ 10-fold higher for DAT but still significantly lower compared to OCT2. This study has shown that several psychostimulants and hallucinogens are substrates for OCTs. Given the extensive cellular uptake of mescaline by the genetically highly polymorphic OCT1, strong interindividual variation in the pharmacokinetics of mescaline might be possible, which could be a reason for highly variable adverse reactions. The involvement of the polymorphic OCT2 in the renal excretion of several psychostimulants could be one reason for individual differences in toxicity.

## Introduction

**FIGURE 1:**
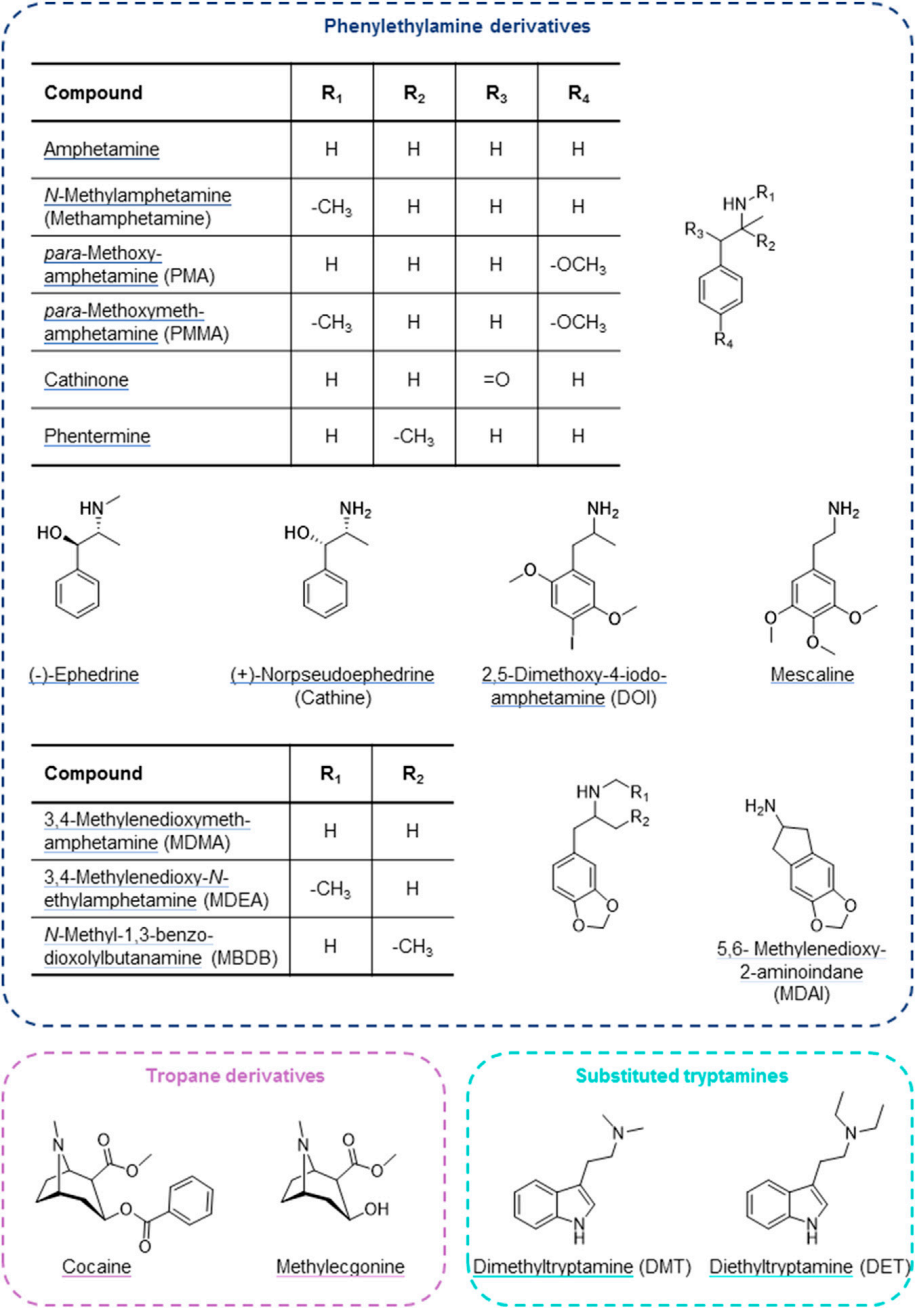
Psychostimulant and hallucinogenic drugs assessed for cell uptake by OCTs and high-affinity monoamine transporters.

Psychostimulants modulate wakefulness and mental performance. They function as indirect sympathomimetics by raising synaptic concentrations of monoamine neurotransmitters through stimulating their release from presynaptic vesicles and/or inhibiting reuptake. Psychostimulants can also interfere with monoaminergic neurotransmitter metabolism and interact with monoaminergic receptors and other targets (Luethi and Liechti, 2020; Reith and Gnegy, 2020). Amphetamine and other phenylethylamine derivatives (Figure 1 top) form a large group of such indirect sympathomimetics. They are used in the treatment of attention deficit hyperactivity disorder and narcolepsy but are also frequently found in illicit drugs (e.g., “speed”, “ecstasy”, “crystal meth”) (Sharma and Couture, 2014; Luethi and Liechti, 2020). Another indirect sympathomimetic is cocaine (Figure 1 bottom left), a tropa-alkaloid and, historically, the first local anesthetic. Its (widely illegal) use as a psychostimulant nowadays far exceeds its therapeutic application in local anesthesia. Psychostimulants are among the most popular drugs of abuse. A related and partially overlapping class of psychoactive substances are the hallucinogens (psychedelics), which alter perception, cognition, and mood. These include tryptamine derivatives, such as the alkaloid dimethyltryptamine (DMT). It is a main constituent of ayahuasca, the plant brew used traditionally by indigenous inhabitants of the Amazon region for spiritual and religious ceremonies. DMT and its diethyl analogue (Figure 1 bottom right) show structural resemblance to the neurotransmitter serotonin and thereby function as agonists at 5-HT_2A_ and related receptors (Nichols, 2016; Luethi and Liechti, 2020). Another traditional hallucinogen is mescaline, a phenethylamine alkaloid found in cacti (Ogunbodede et al., 2010; Nichols, 2016; Luethi and Liechti, 2020). It is a partial agonist at 5-HT_2A_ and 5-HT_2B_ receptors and a full agonist at the 5-HT_2C_ receptor (Dinis-Oliveira et al., 2019).

Many psychoactive substances are substrates or inhibitors of the noradrenaline (norepinephrine) transporter (NET), the dopamine transporter (DAT), and/or the serotonin transporter (SERT) (Luethi and Liechti, 2020). These high-affinity transport proteins are expressed at presynaptic neurons, where they mediate the reuptake of monoamine neurotransmitters from the synaptic cleft to terminate synaptic signal transmission and for recycling (Torres et al., 2003). They are members of the large Solute Carrier (SLC) superfamily and coded for by the genes *SLC6A2* (NET), *SLC6A3* (DAT), and *SLC6A4* (SERT).

Organic cation transporters (OCTs) are also SLCs with a broad, partially overlapping substrate spectrum that is predominantly comprised of hydrophilic, organic cationic substances (including monoamine neurotransmitters as well as many drugs) (Busch et al., 1998; Gründemann et al., 1998; Wu et al., 1998; Koepsell et al., 2007). OCT1 (*SLC22A1*) and, to a lesser extent, OCT3 (*SLC22A3*) are expressed on the sinusoidal membrane of hepatocytes, where they mediate cellular uptake for hepatic metabolism (Nishimura and Naito, 2005; Nies et al., 2009). A high degree of genetic variation exists for *SLC22A1*, and several of these variants strongly impact transporter expression and function (Koepsell et al., 2007; Seitz et al., 2015). This may affect the pharmacokinetics of compounds that are substrates of OCT1, as has been shown, for example, for the opioid analgesics morphine and *O*-desmethyltramadol (Tzvetkov et al., 2011; Tzvetkov et al., 2013; Venkatasubramanian et al., 2014; Stamer et al., 2016), the antimalarial prodrug proguanil (Matthaei et al., 2019), the anti-asthma drug fenoterol (Tzvetkov et al., 2018), sumatriptan that is used for the treatment of migraine (Matthaei et al., 2016), and, to a minor extent, for the antidiabetic drug metformin (Tzvetkov et al., 2009; Yee et al., 2018). The psychoactive substances studied here (Figure 1) were selected based on physicochemical properties (organic cations with *pK*_*a*_ > 8.4 and relatively hydrophilic substances with a log*D*_pH 7.4_ < 2; Table 1) that make them potential substrates for OCTs. Consequently, their pharmacokinetics could potentially be affected by OCT polymorphism as well. OCT2 (*SLC22A2*) is mainly found on the basolateral membrane of kidney epithelial cells (Motohashi et al., 2002; Motohashi et al., 2013). Together with multidrug and toxin extrusion protein 2 kidney-specific (MATE2-K, *SLC47A2*), an efflux transporter expressed on the brush-border membrane of the proximal tubule, it mediates transport across the epithelium for renal excretion (Motohashi et al., 2013). *SLC22A2* variants are less frequent compared to the gene coding for OCT1, and only a few affect OCT2 expression or function. The most frequent of these is Ala270Ser, which causes a moderate decrease in OCT2 activity (Zolk et al., 2009). As many psychoactive substances are structurally related to the neurotransmitters and OCT substrates noradrenaline, dopamine, and serotonin and have physicochemical properties in line with typical OCT substrates, their pharmacokinetics may be determined by OCTs and influenced by OCT1 (and possibly OCT2) polymorphism.

**TABLE 1:** Physicochemical properties of investigated psychoactive compounds (predicted using MarvinSketch, version 19.8, ChemAxon, Budapest, Hungary).

Test compound	LogD_pH__7.4_	pK_a_	% Positively charged at pH 7.4
Amphetamine	−0.67	10.01	99.76
Methylamphetamine	−0.44	10.21	99.85
PMA	−0.85	10.04	99.77
PMMA	−0.52	10.03	99.76
Cathinone	0.79	7.55	58.59
Phentermine	−0.55	10.25	99.78
(-)-Ephedrine	−0.78	9.53	99.26
Cathine	−1.05	9.37	98.94
DOI	0.02	9.90	99.69
Mescaline	−1.37	9.77	99.58
MDMA	−0.76	10.14	99.82
MDEA	−0.46	10.22	99.85
MBDB	−0.34	10.28	99.87
MDAI	−1.33	9.96	99.73
Cocaine	0.82	8.85	96.54
Methylecgonine	−1.86	9.04	97.76
DMT	0.17	9.55	99.29
DET	0.39	10.08	99.79

Although mainly expressed in peripheral tissues, OCT2 and OCT3 are also found on postsynaptic neurons (and OCT3 in astrocytes) predominantly in aminergic regions of the central nervous system. There, they may be involved in reuptake of monoamine neurotransmitters in brain areas lacking the high-affinity transporters, at distance from the aminergic nerve endings, or as an alternative when the high-affinity transporters are saturated or inhibited (Wu et al., 1998; Vialou et al., 2008; Bacq et al., 2012; Couroussé and Gautron, 2015). OCT2 appears to be involved in the uptake of noradrenaline and serotonin in particular, while OCT3 was found to be more strongly responsible for dopamine clearance (Vialou et al., 2008; Bacq et al., 2012). Interestingly, it has also been shown that amphetamines can induce neurotransmitter release through OCT3, which is capable of bi-directional transport (Mayer et al., 2018; Mayer et al., 2019). Thus, OCTs may not only determine the pharmacokinetics of psychoactive drugs but appear to be also involved in their actions.

Given the potential dual role of OCTs with respect to psychoactive drugs and the current lack of understanding of the pharmacokinetics and pharmacogenetics for these compounds, we characterised the transmembrane transport by polyspecific OCTs as well as high-affinity monoamine reuptake transporters. Of particular interest are those psychostimulants that are stereoisomers of one another (ephedrine, norephedrine, their enantiomers and diastereomers), because the impact of stereospecificity on membrane transport is as yet not well understood but previous results suggest partially strong enantiopreferences (Jensen et al., 2020).

## Materials and Methods

### Test Compounds

The psychoactive compounds studied here were selected based on their physicochemical properties that would make them likely substrates for OCTs. Selection criteria included hydrophilicity (logD at pH 7.4 of less than 2), at least 90% positively charged at physiological pH (pK_a_ > 8.4), and molecular mass not higher than 500 Da. The reasons for these were that lipophilic compounds permeate membranes mostly by diffusion, while membrane transport is mostly relevant for more hydrophilic compounds, as well as the observation that typical OCT1 substrates are usually positively charged and of low to moderate size. Cathinone (pK_a_ of 7.55) did not meet our selection criteria but was nonetheless included due to a low renal elimination (2–7% unchanged in urine) and, consequently, high rate of metabolism which, if taking place in the liver, might depend on hepatic uptake via OCT1 (Kalix and Braenden, 1985; Toennes and Kauert, 2002). Ranitidine-d6 was purchased from Toronto Research Chemicals (Toronto, Canada) and Tulobuterol from Santa Cruz Biotechnology (Darmstadt, Germany); all other test compounds and internal standards were bought from Sigma-Aldrich (Taufkirchen, Germany).

### Generation of Transporter-Overexpressing Cell Lines

Transport experiments were done using HEK293 cells stably transfected to overexpress OCT1*1 (wild-type), OCT1*2 (M420del), OCT1*3 (R61C), OCT1*4 (G401S), OCT1*5 (M420del, G465R), OCT1*6 (C88R, M420del), OCT1*7 (S14F), OCT1*8 (R488M), as well as wild-type OCT2, OCT3, NET, DAT, SERT, or MATE2-K. All cell lines were generated using the Flp-In system (Thermo Fisher Scientific, Darmstadt, Germany) as previously described (Saadatmand et al., 2012; Seitz et al., 2015; Chen et al., 2017), except for the OCT3-overexpressing HEK293 cells that were a kind gift from Drs. Koepsell and Gorbulev (University of Würzburg, Germany). The cells were kept in culture for no more than 30 passages.

**FIGURE 2:**
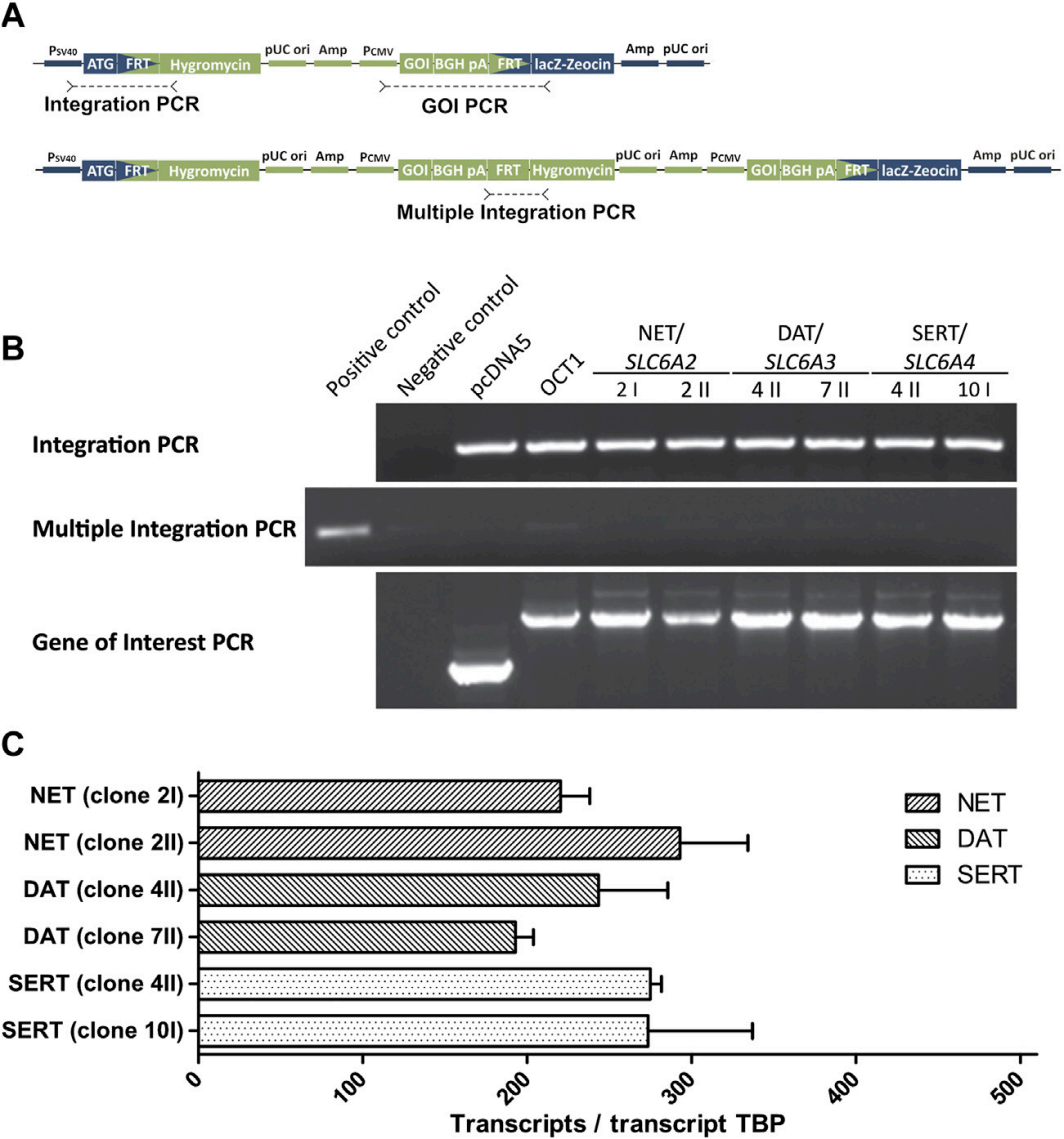
Validation of HEK293 cell clones overexpressing monoamine neurotransmitter transporters **(A)** Schematic representation of the expression plasmid pcDNA5 (green) and the host cell line genome (blue) at the FRT site showing the target positions of the three conducted PCRs **(B)** Results of the three validation PCRs that show a successful integration (Integration PCR) for all newly created cell clones that overexpress the high-affinity monoamine transporters. The absence of amplicons in the Multiple Integration PCR indicate a single integration of the pcDNA5 plasmid. The correctness of amplified genes in the Gene of Interest (GOI) PCR was validated by Sanger sequencing **(C)** Quantitative real-time PCR results to confirm comparable overexpression of monoamine transporters, shown as transcripts per transcript of the TATA-binding protein. Only one cell clone was selected per transporter for experiments.

The high-affinity monoamine transporters were also stably transfected into HEK293 cells by use of the Flp-In system (Thermo Fisher Scientific, Darmstadt, Germany). Coding sequences of *SLC6A2* (NET), *SLC6A3* (DAT), and *SLC6A4* (SERT) were obtained from Source BioScience (Nottingham, United Kingdom; pBluescriptR:SLC6A2) or Addgene (Watertown, MA, United States; pcDNA3.1-hDAT was a gift from Susan Amara, Addgene plasmid # 32810, http://n2t.net/addgene:32810, RRID:Addgene_32810 and hSERT pcDNA3 was a gift from Randy Blakely, Addgene plasmid # 15483, http://n2t.net/addgene:15483, RRID:Addgene_15483 (Ramamoorthy et al., 1993)). After sequence correction and cloning into the pcDNA5 vector, generation and characterization of the cell lines were carried out as described before for the above-mentioned cell lines (Saadatmand et al., 2012; Seitz et al., 2015). Genomic integration was validated for two independent cell clones by three polymerase chain reactions (PCR; Figure 2) to verify proper integration (integration PCR) and exclude multiple integration (multiple integration PCR). The presence of the gene of interest was verified by Sanger sequencing of the product of the third PCR (gene-of-interest PCR) after gel extraction (Figure 2). Overexpression of monoamine transporters was compared between cell clones by TaqMan® gene expression assays (Thermo Fisher Scientific, Darmstadt, Germany; Figure 2). Functional validation of newly generated cell clones was performed using noradrenaline and serotonin as probe drugs and one clone for each transporter was chosen for further transport studies.

### *In vitro* Cellular Uptake Experiments

The HEK293 cells were cultered in DMEM medium supplemented with 10% (v/v) fetal bovine serum as well as penicillin (100 U/ml) and streptomycin (100 μg/ml) obtained from Thermo Fisher Scientific (Darmstadt, Germany). Cells were seeded on 12-well plates coated with poly-d-lysine (Sigma-Aldrich, Taufkirchen, Germany) 48 h before the transport experiments and incubated at 37°C, 95% relative humidity, and 5% CO_2_. Cell lines overexpressing MATE2-K were incubated with 30 mM NH_4_Cl in HBSS+ (10 mM HEPES in HBSS, pH 7.4; Thermo Fisher Scientific, Darmstadt, Germany) for 30 min prior to the assay to invert the direction of transport. All cell lines were washed with 37°C HBSS+ and subsequently incubated with the pre-warmed substrate in HBSS+ at 37°C. The time points for measuring substrate uptake were 1 min for MATE2-K and 2 min for the other SLCs. The uptake rate was experimentally determined to be linear for at least 10 min for OCT1*1. It was assumed to be linear for the other transporters as well, based on previous experience with these expression systems. The reaction was stopped by adding ice-cold HBSS+, and the cells were washed twice with ice-cold HBSS+ before lysis with 80% acetonitrile (LGC Standards, Wesel, Germany) including an internal standard. Subsequently, the intracellular substrate accumulation was determined using LC-MS/MS.

### Concentration Analyses

Intracellular accumulation was measured by HPLC-MS/MS using a Shimadzu Nexera HPLC system with a LC-30AD pump, a SIL-30AC autosampler, a CTO-20AC column oven, and a CBM-20A controller (Shimadzu, Kyoto, Japan). Separation was done on a Brownlee SPP RP-Amide column (4.6 × 100 mm inner dimension with 2.7 μm particle size) with a C18 pre-column. The aqueous mobile phase contained 0.1% (v/v) formic acid and either 3% (v/v) organic additive (acetonitrile:methanol 6:1 (v/v)) for methylecgonine, 8% for amphetamine, methylamphetamine, cathinone, cathine, (-)-ephedrine, mescaline, MDAI, and DMT, or 20% for PMA, PMMA, DOI, phentermine, MDMA, MDEA, MBDB, cocaine, and DET. Chromatography was done at a flow rate of 0.3 ml/min. For detection, an API 4000 tandem mass spectrometer (AB SCIEX, Darmstadt, Germany) was used in MRM mode. The analytes, corresponding internal standards, and detection parameters are listed in the Supplementary Table S1. Peak integration and quantification of the analytes was done using the Analyst software (Version 1.6.2, AB SCIEX, Darmstadt, Germany) and determined by simultaneous measurement of standard curves with known concentrations.

### Calculations

For the screenings, cellular uptake measured in cell lines overexpressing the respective transporter was divided by the uptake measured in an empty vector control cell line to calculate normalised ratios to enable comparisons between test compounds. For studying transport kinetics, the net transport mediated by the overexpressed transporters was calculated by subtracting the cellular uptake measured in an empty vector control cell line from the uptake in cell lines overexpressing the respective transporter. The parameters *K*_*m*_ and *v*_max_ were estimated by regression analysis using the Michaelis-Menten equation (GraphPad Prism version 5.01 for Windows, GraphPad Software, La Jolla, CA, United States). Means and standard errors were calculated from individual *K*_*m*_ and *v*_max_ values of at least three independent experiments. The kinetic parameters *v*_max_ and *K*_*m*_ were tested for statistical significance over empty vector control cells using Student’s t-test with an alpha value of 0.05.

## Results

### Screening of Transport Activity at OCTs, Monoamine Transporters, and MATE2-K

**FIGURE 3:**
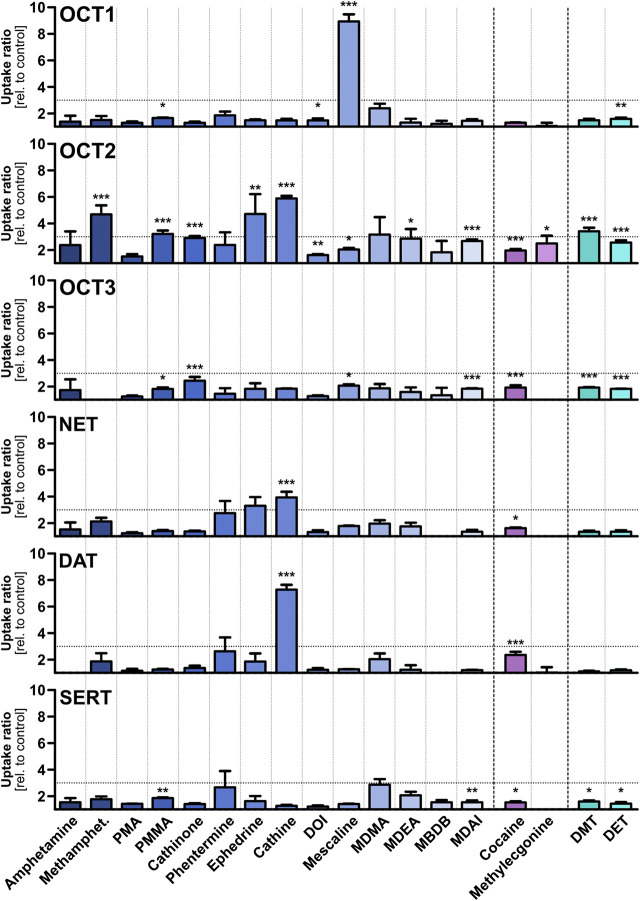
Transport of different psychostimulant and hallucinogenic substances at a concentration of 1 µM by OCTs and high-affinity monoamine transporters, shown as the ratios of uptake after 2 min in transporter-transfected cells over empty vector control cells. Shown are the mean values of ≥3 independent experiments +SEM. The horizontal dotted line indicates an uptake ratio of 3, which was set as the minimum threshold for more detailed characterisation. Statistical significance over empty vector control cells was determined using Student’s t-test with *p < 0.05, **p < 0.01, and ***p < 0.001.

Eighteen psychostimulants and hallucinogens were initially screened for their potential to be substrates for different polyspecific OCTs and high-affinity monoamine neurotransmitter transporters (Figure 3), as well as for the efflux transporter MATE2-K (Supplementary Figure S1). The compounds were assessed at a concentration of 1 μM, because it is unlikely that low-affinity transport at higher concentrations may have any medical relevance and the relative contribution of carrier-mediated transport over passive diffusion is significantly greater at lower compared to higher substrate concentrations, as was previously shown for morphine (Tzvetkov et al., 2013). Although the test compounds were selected based on physicochemical properties that are in accordance with those of typical OCT substrates, OCT1 showed high transport activity at this concentration only for mescaline. A cellular uptake in transporter-transfected cells of at least 3-fold higher than in non-overexpressing control cells was selected as the threshold for further studies, as this ratio is suitable to distinguish substrates from non-substrates. Cellular uptake of mescaline was more than 8-fold higher in OCT1-overexpressing cells, which was the highest transport activity that was observed altogether in this study. Interestingly, mescaline was not transported much at 1 µM by any of the other transporters. In contrast to the substrate-specific but very strong transport activity exhibited by OCT1, moderate (4- to 6-fold) cellular uptake by OCT2 was seen for methamphetamine, (-)-ephedrine, and cathine ((+)-norpseudoephedrine) and approximately 3-fold for *para*-methoxymethamphetamine (PMMA) and DMT. OCT3 and MATE2-K (Supplementary Figure S1) showed little or no transport activity with any of the 18 psychoactive compounds studied here at 1 µM. Our observation, that amphetamine does not appear to be a substrate of OCT3, is in accordance with previous reports (Zhu et al., 2010).

The OCTs are known as low-affinity, high-capacity solute carriers with a very broad substrate spectrum that comprises structurally diverse compounds. In contrast, the monoamine neurotransmitter reuptake transporters NET, DAT, and SERT show high affinities to their respective endogenous substrates and a more narrow substrate profile than the OCTs. Cathine was transported modestly (4-fold) by NET and higher (7-fold) by DAT. No notable transport activity was observed for the other compounds, and none by SERT altogether. Cathine and (-)-ephedrine (as well as their stereoisomers) have been described previously as substrates for NET and DAT in *vitro* experiments with very different setup (Rothman et al., 2003). The slightly higher (albeit still low) uptake of PMA and PMMA by SERT compared to DAT is in line with literature reports that substitution in *para*-position of the phenyl ring of amphetamine derivatives shifts substrate preference toward SERT (Simmler et al., 2014).

### Concentration-dependent Transport of Mescaline by OCT1 Genetic Variants

**FIGURE 4:**
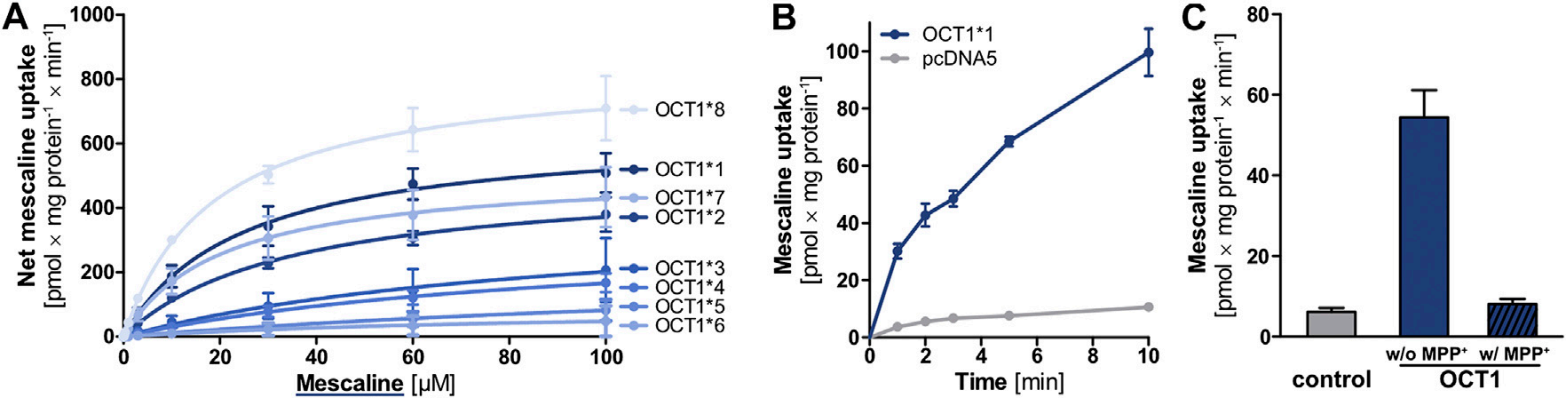
**(A)** Transport of mescaline at different concentrations by OCT1*1 (wild-type) and genetic variants, shown as the cellular uptake in transporter-overexpressing cells with substracted uptake in non-overexpressing control cells. Shown are the mean values of ≥3 independent experiments ±SEM. K_m_ and v_max_ values are given in Table 2. **(B)** Time-dependent uptake of 1 µM mescaline in OCT1*1-overexpressing (blue) and non-overexpressing control (pcDNA5, gray) cells, shown as the mean values of 3 independent experiments ±SEM **(C)** Mescaline transport by OCT1 could be completely inhibited by 1 mM MPP^+^ to values not significantly different from the unspecific cellular uptake observed in empty vector-transfected cells (control).

Mescaline was found in our substrate screenings to be strongly transported by OCT1 and, therefore, it was studied in more detail. Given the high degree of genetic polymorphism and the large differences in transporter activity and expression for some variants, cellular uptake of mescaline was not only characterised for wild-type (OCT1*1) but for OCT1 variants *2 to *8 as well. OCT1*1 transported mescaline with a *K*_*m*_ of 24.3 ± 6.3 µM and a *v*_max_ of 642 ± 57 pmol × mg protein^−1^ × min^−1^ (Figure 4A, Table 2). Time-dependent uptake of 1 µM mescaline showed a faster uptake rate within the first minute of incubation and a constant, linear uptake rate for 2 to at least 10 min (Figure 4B). The apparently more rapid initial uptake rate is likely a result of high-affinity binding to OCT1, but a short-lived more rapid transport might also be possible. The constant transport rate after 2 min of incubation might be the more relevant transport rate for pharmacokinetics because the exposure of the liver and other organs to drugs and other substances usually occurs for several hours. Mescaline uptake could be completely inhibited by the competitive OCT1 inhibitor 1-methyl-4-phenylpyridinium (MPP^+^; Figure 4C). The *K*_*m*_ was slightly higher and the *v*_max_ slightly lower for *2, which is analogous to literature data on reduced transport activity for *2 (Seitz et al., 2015; Koepsell, 2020). This was even more pronounced (*K*_*m*_ of 93.6 ± 110.8 and 98.2 ± 46.7 µM; *v*_max_ of 391 ± 266 and 329 ± 92 pmol × mg protein^−1^ × min^−1^) for *3 and *4, which are known to have strongly reduced transport activity (Seitz et al., 2015; Koepsell, 2020). For the variants *5 and *6 that result in impaired translocation to the plasma membrane (Seitz et al., 2015), very low transport activity was observed. Consequently, *K*_*m*_ and *v*_max_ values could not be reliably calculated. OCT1*7 exhibited a similar *K*_*m*_ and a modestly reduced *v*_max_ than OCT1*1. OCT1*8, on the other hand, showed a higher *v*_max_ than the wild-type, which has been reported previously for a number of substrates as well (Seitz et al., 2015; Koepsell, 2020). To summarise, transport activity of mescaline was slightly lower than wild-type OCT1 in variants *2 and *7, more drastically reduced in *3 and *4, and lowest in *5 and *6, while *8 showed a moderately higher *v*_max_ than wild-type OCT1.

**TABLE 2:** Kinetic parameters for the transport of mescaline by different OCT1 genetic variants.

Variant	K_m_ [µM]	v_max_ [pmol × mg protein^−1^ × min^−1^]
OCT1*1 (WT)	24.3 (±6.3)	641.7 (±57.1)
OCT1*2 (M420del)	34.7 (±7.4)	500.7 (±42.1)
OCT1*3 (R61C)	93.6 (±110.8)	390.7 (±265.8)
OCT1*4 (G401S)	98.2 (±46.7)	329.4 (±91.6)
OCT1*5 (M420del, G465R)	Not determinable	Not determinable
OCT1*6 (M420del, C88R)	Not determinable	Not determinable
OCT1*7 (S14F)	20.2 (±7.9)	514.6 (±63.8)
OCT1*8 (R488M)	18.6 (±3.7)	837.2 (±51.5)

### Concentration-dependent Transport of Methamphetamine, PMMA, (-)-Ephedrine, Cathine, and DMT by OCT2 Wild-type and A270S Variant

**FIGURE 5:**
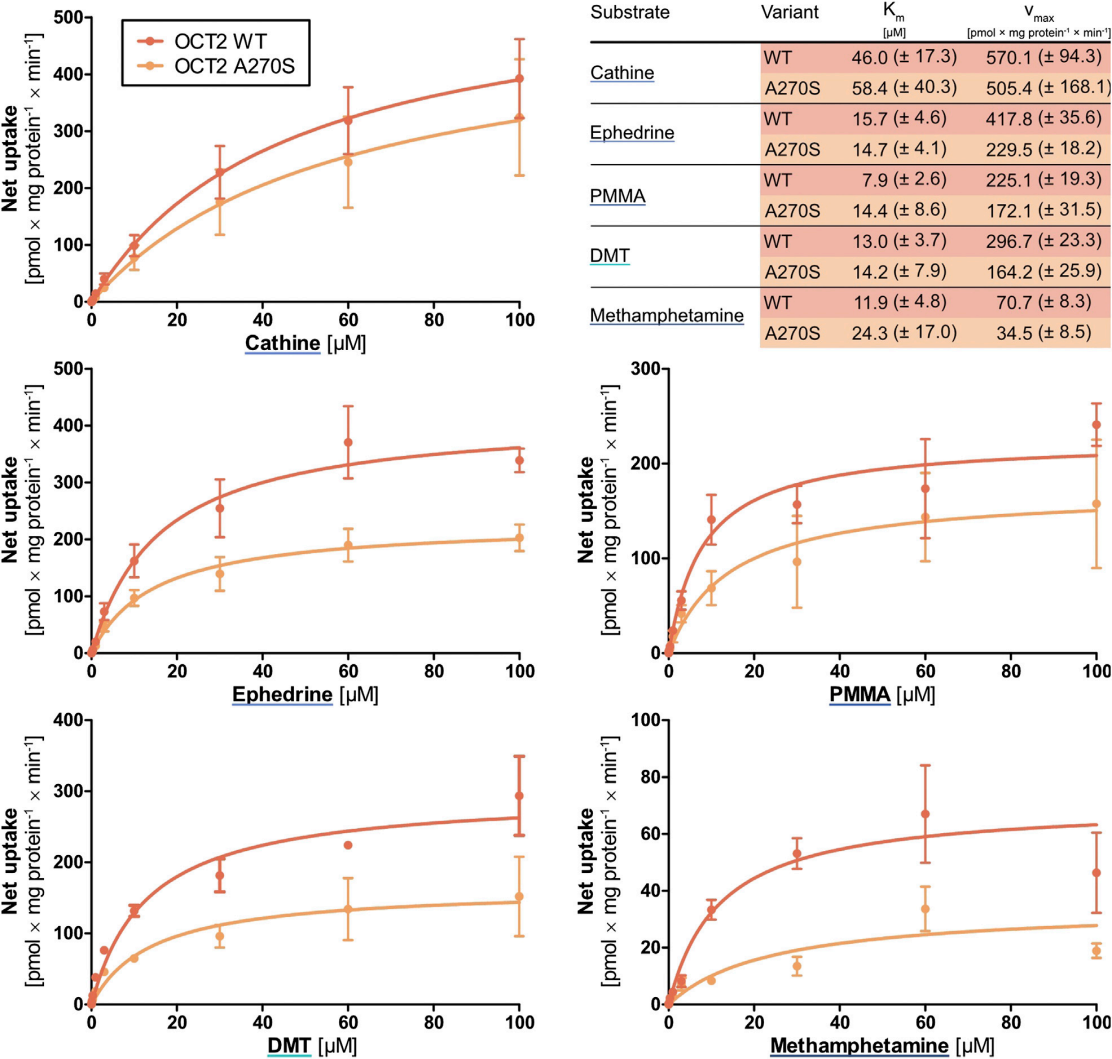
Transport of cathine, (-)-ephedrine, PMMA, DMT, and methamphetamine at different concentrations by wild-type OCT2 (red) and the variant A270S (orange), shown as the cellular uptake in transporter-overexpressing cells with substracted uptake in non-overexpressing control cells. Shown are the mean values of ≥3 independent experiments ±SEM.

Whereas only mescaline appeared to be a substrate for OCT1, transport *via* OCT2 was seen for methamphetamine, PMMA, (-)-ephedrine, cathine, and DMT. These compounds were subsequently assessed in greater detail (Figure 5). For methamphetamine, the v_max_ for wild-type OCT2 was only 70.7 ± 8.3 pmol × mg protein^−1^ × min^−1^, whereas it was between 225 and 570 pmol × mg protein^−1^ × min^−1^ for the other four compounds. The *K*_*m*_ values were around 10 µM except for cathine (46.0 ± 17.3 µM). For the A270S variant, the *v*_max_ values were slightly to moderately lower (except for PMMA) and the *K*_*m*_ values either similar ((-)-ephedrine and DMT) or up to 4-fold higher (methamphetamine, PMMA, cathine) compared to wild-type OCT2, in agreement with literature reports that the A270S exchange can lead to a moderate decrease in OCT2 activity (Zolk et al., 2009).

### Concentration-dependent Transport of Cathine by NET and DAT

**FIGURE 6:**
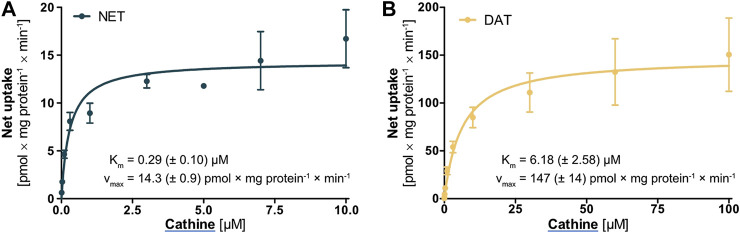
Transport of cathine at different concentrations by **(A)** NET and **(B)** DAT, shown as the cellular uptake in transporter-overexpressing cells with substracted uptake in non-overexpressing control cells. Shown are the mean values of ≥3 independent experiments ±SEM.

Cathine was the only compound for which notable cellular uptake was observed by the high-affinity monoamine transporters NET and DAT. Further characterisation and a comparison between NET and DAT revealed that the *K*_*m*_ was 21-fold and the *v*_max_ 10-fold higher for DAT (Figure 6). Yet, both *K*_*m*_ and *v*_max_ were still significantly lower compared to OCT2, in line with the general description of NET and DAT as high-affinity and low-capacity transporters.

## Discussion

In this study, three groups of psychostimulants and hallucinogens (14 phenylethylamine derivatives, the tropanes cocaine and methylecgonine, and the substituted tryptamines dimethyl- and diethyltryptamine) were assessed for their substrate properties for OCTs as well as for high-affinity monoamine transporters. OCTs are known to have a very broad substrate profile that comprises many different structural classes. It is therefore surprising that only relatively few of the 18 psychoactive compounds studied here were moderate or good OCT substrates, especially because these were selected based on physicochemical properties that were in accordance with those of typical OCT substrates. Other transporters, such as OCTN1 and OCTN2, the proposed H^+^-organic cation antiporter, or ATP-binding cassette efflux transporters might potentially be more relevant for some of the tested psychoactive compounds.

Only mescaline was transported significantly at 1 µM by OCT1, and that this was the highest transport activity observed here altogether. With a pK_a_ of 9.77, a logD_pH 7.4_ of -1.37, and a molecular mass of 211.3 g/mol, its physicochemical properties are not significantly different from those of the other compounds (Table 1). It is thus reasonable to wonder what properties make mescaline the only substrate at this concentration compared to the 17 other compounds studied here. Possible explanations are not evident from its chemical structure, as it is an amphetamine derivative structurally relatively similar to many of the other phenylethylamines.

**FIGURE 7:**
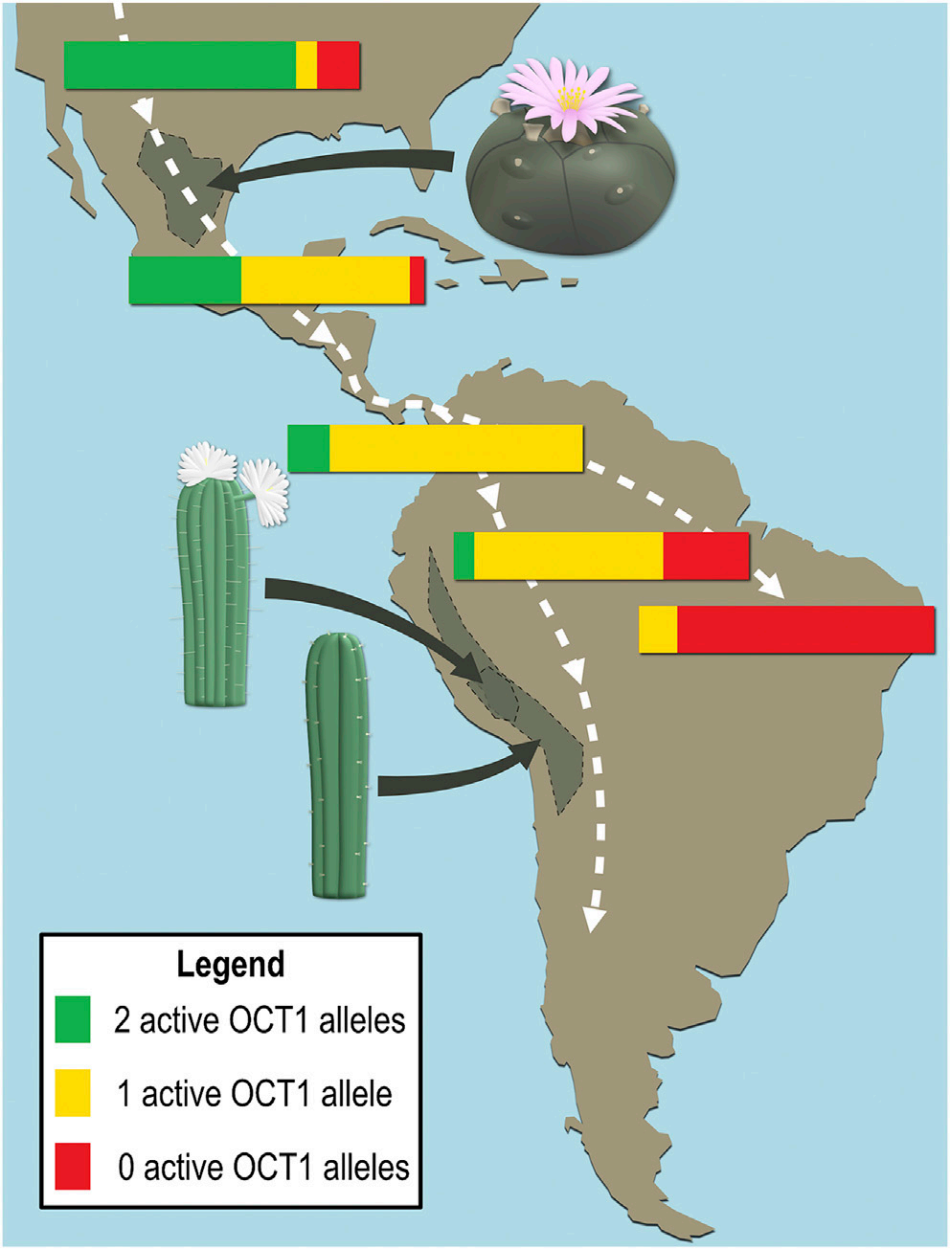
Schematic representation of the frequency distributions of active and inactive OCT1 alleles in local populations and natural habitats of the high mescaline-containing cacti *Lophophora williamsii* (peyote), *Echinopsis pachanoi* (San Pedro), and *Echinopsis peruvianus* (Peruvian torch). The white dashed lines broadly illustrate the migration pattern during the first population of the continent by humans.

Mescaline is an alkaloid biosynthesised from tyrosine in different cacti, where it is found at concentrations of 0.05–4.7% by dry weight (Ogunbodede et al., 2010). *Lophophora williamsii* (peyote cactus) and several *Echinopsis* species (e.g., *Echinopsis pachanoi* and *Echinopsis peruvianus*, also known as the San Pedro and the Peruvian torch cacti) have a long-standing use in religious ceremonies and traditional medicine of South American indigenous populations. The hallucinogenic effects of these cacti were attributed to their relatively high mescaline contents (Ogunbodede et al., 2010; Dinis-Oliveira et al., 2019; da Silveira Agostini-Costa, 2020). Interestingly, OCT1 deficiency or reduced activity is more frequently found in Central and South American populations than in most other parts of the world and the prevalence of inactive alleles generally increases further south on the American continent (Figure 7) (Seitz et al., 2015). It is likely that OCT1 deficiency was somehow advantageous, e.g., in connection with dietary ingredients that are OCT1 substrates (or perhaps mescaline?), and inactive alleles thus dominated as the first human inhabitants of the continent migrated south.

Typical mescaline dosages are in the range of 170–400 mg, which induce a psychedelic state that may involve visual hallucinations, altered perception, synesthaesia, and euphoria. The lifetime prevalence of mescaline use over the past 3 decades was estimated to be between 3–4% in the United States (Dinis-Oliveira et al., 2019; Johnson et al., 2019). Being a high-affinity partial agonist for the 5-HT_2A_ receptor, potential therapeutic uses for mescaline were proposed for disorders associated with serotonin deficiency, such as addiction, anxiety, and depression (Kyzar et al., 2017; Johnson et al., 2019). Based on the key finding of this study, that mescaline is a strong substrate of the genetically highly polymorphic OCT1, large interindividual variations in mescaline pharmacokinetics might be possible. This could lead to intoxication and other adverse effects due to decreased elimination in carriers of alleles with reduced or absent OCT1 activity (e.g., OCT1 variants *2 to *6, which are particularly common in European populations, or OCT1*7 that is frequently found in Africans and Afro-Americans (Seitz et al., 2015)). However, a substance being identified as OCT1 substrate in vitro may not necessarily be affected by OCT1 genetic polymorphism in vivo, as illustrated by the example of the indirect sympathomimetic compound tyramine (Rafehi et al., 2019). Thus, the effects of OCT1 genotype on mescaline should be studied in vivo and its clinical implications taken into consideration when developing therapeutic interventions involving mescaline.

Another key result of this study was that methamphetamine, PMMA, (-)-ephedrine, cathine, and DMT were substrates of OCT2 and that their transport was moderately reduced in the A270S variant. OCT2 is strongly expressed in the kidneys, where it contributes to transepithelial transport of usually hydrophilic substances and thereby renal elimination. Cathine was excreted unchanged in urine to 46–65% in four healthy volunteers and the renal elimination was reported to be 70% for (-)-ephedrine and 30–54% for methamphetamine (Toennes and Kauert, 2002; www.dosing.de and www.drugbank.ca, both accessed on September 16, 2020). The reduced transport by the A270S variant of OCT2 might thus possibly result in a decreased elimination of these compounds. Besides variation due to inherited polymorphisms, variation in renal elimination of these psychostimulants may additionally arise from drug-drug interactions or conditions associated with increased blood concentrations of endogenous organic cations. DMT, on the other hand, is extensively metabolised and excreted unchanged in urine only to a very low extent (e.g., 0.16% following intramuscular administration) (Sitaram et al., 1987; Barker, 2018). OCT2 polymorphism is thus unlikely to have any significant effects on DMT pharmacokinetics but might still influence local concentrations of DMT as well as of methamphetamine, PMMA, (-)-ephedrine, and cathine in the central nervous system due to OCT2 expression in postsynaptic neurons.

OCT1 and OCT2 polymorphism is not the only form of genetic variation that may affect the above-mentioned compounds. Metabolising enzymes and target receptors may also be polymorphic. A few examples regarding the pharmacogenetics of these compounds are given in Table 3. A good example for discussing the general importance of genetic polymorphism is MDMA, as this psychostimulant has been studied in greater detail. MDMA is widely used as the recreational drug “ecstasy” but therapeutic use for the treatment of posttraumatic stress disorder has also been proposed (Mithoefer et al., 2011; Mithoefer et al., 2013; Amoroso and Workman, 2016; Mithoefer et al., 2016). It is a substrate of the polymorphic enzymes cytochrome P450 (CYP) 2C19, 2B6, and 1A2, which catalyze the conversion to 3,4-methylenedioxyamphetamine. Carriers of genetic variants that result in increased activity of these enzymes showed higher metabolism and CYP2C19 poor metabolisers had greater cardiovascular effects in response to MDMA consumption (Schindler et al., 2014; Vizeli et al., 2017). Poor metabolisers for the highly polymorphic CYP2D6 also showed higher cardiovascular responses, but only to a minor extent due to the inhibition of CYP2D6 (Schmid et al., 2016). Based on in vitro data, the effect of CYP2D6 polymorphism was previously predicted to be higher (La Torre et al., 2012). MDMA has a basic secondary amine group that is protonated to 99.8% at physiological pH (Table 1). It would thus require a transport mechanism for efficient passage across cell membranes and into hepatocytes for metabolism. Our results suggest that OCTs only contribute to a minor extent. Although MDMA is not a good OCT substrate, its metabolites might possibly be (as we had previously shown analogously for different opioids, where their more hydrophilic metabolites were better OCT substrates (Meyer et al., 2019)). For example, the main metabolites 3,4-dihydroxyamphetamine and 3,4-dihydroxymethamphetamine are more hydrophilic than MDMA and might thus potentially be better OCT substrates, as they would likely rely more strongly on transport mechanisms to traverse cell membranes. However, the present study has shown that substrate specificity cannot always be predicted based on physicochemical properties alone. Although a number of contributors to the serotonergic system are polymorphic, significant variation in MDMA effects were not seen in healthy humans (Vizeli et al., 2019). NET polymorphism also showed only minor effects on the cardiovascular response to MDMA in clinical studies (Vizeli et al., 2018). To summarise this, genetic polymorphism significantly determines the pharmacokinetics but not so much the pharmacodynamics of MDMA (and possibly of other psychostimulants as well).

**TABLE 3:** Pharmacogenetics of methamphetamine, PMMA, (-)-ephedrine, cathine, mescaline, and DMT (this list is not exhaustive).

Test compound	Substrate of			Polymorphic targets^a^	References
	OCT1	OCT2	Polymorphic enzymes^a^		
Methamphetamine	−	++	CYP2D6, FMO3	TAAR1, VMAT2, MAO	Cashman et al. (1999), Eiden and Weihe (2011), Miller 2011, Smith et al. (2012), and Matsusue et al. (2018)
PMMA	−	+	CYP2D6	TAAR1, 5-HT_2A_	Simmler et al. (2014), and Vevelstad et al. (2017)
(-)-Ephedrine	−	++		*ß*_2_-adrenoceptor	Rao et al. (2019)
Cathine	−	+++		*ß*_1_- and *a*_2A_-adrenoceptors	Adeoya-Osiguwa and Fraser (2007)
Mescaline	+++	−	Possibly MAO	5-HT_2A_,5-HT_2C_,TAAR1	Spector (1961), Lerer et al. (2001), Mulder et al. (2007), Kling et al. (2008), Hoekstra et al. (2010), Rickli et al. (2015), and Dinis-Oliveira et al. (2019)
DMT	−	+	MAO-A	5-HT_2A_,5-HT_2C_,TAAR1	Keiser et al. (2009), Rickli et al. (2016), and Barker (2018)

^a^ Abbreviations: 5-HT, 5-hydroxytryptamine; CYP2D6, cytochrome P450 subtype 2D6; FMO3, Flavin-containing monooxygenase 3; MAO, monoamine oxidase; TAAR1, trace amine-associated receptor 1, VMAT2, vesicular monoamine transporter 2

A concept that has so far not received much attention is stereoselectivity in membrane transport. Recent results from our laboratory have shown that transmembrane transport of adrenergic drugs by OCTs can show strong enantiospecificity (Jensen et al., 2020). The phenylethylamine derivatives cathine (also referred to as (+)-norpseudoephedrine) and (-)-ephedrine that were assessed in this study are chiral compounds and structurally very closely related. If it were not for the methyl substitution at the amino group (Figure 1), both compounds would be stereoisomers of one another. With this in mind, it appears astonishing that cathine was found to be a good substrate of DAT whereas (-)-ephedrine was not, despite their close structural resemblance. Whether this difference in transport was due to the opposite steric orientation of the hydroxyl group or due to the methyl substitution at the amino group cannot be deduced from this study.

**FIGURE 8:**
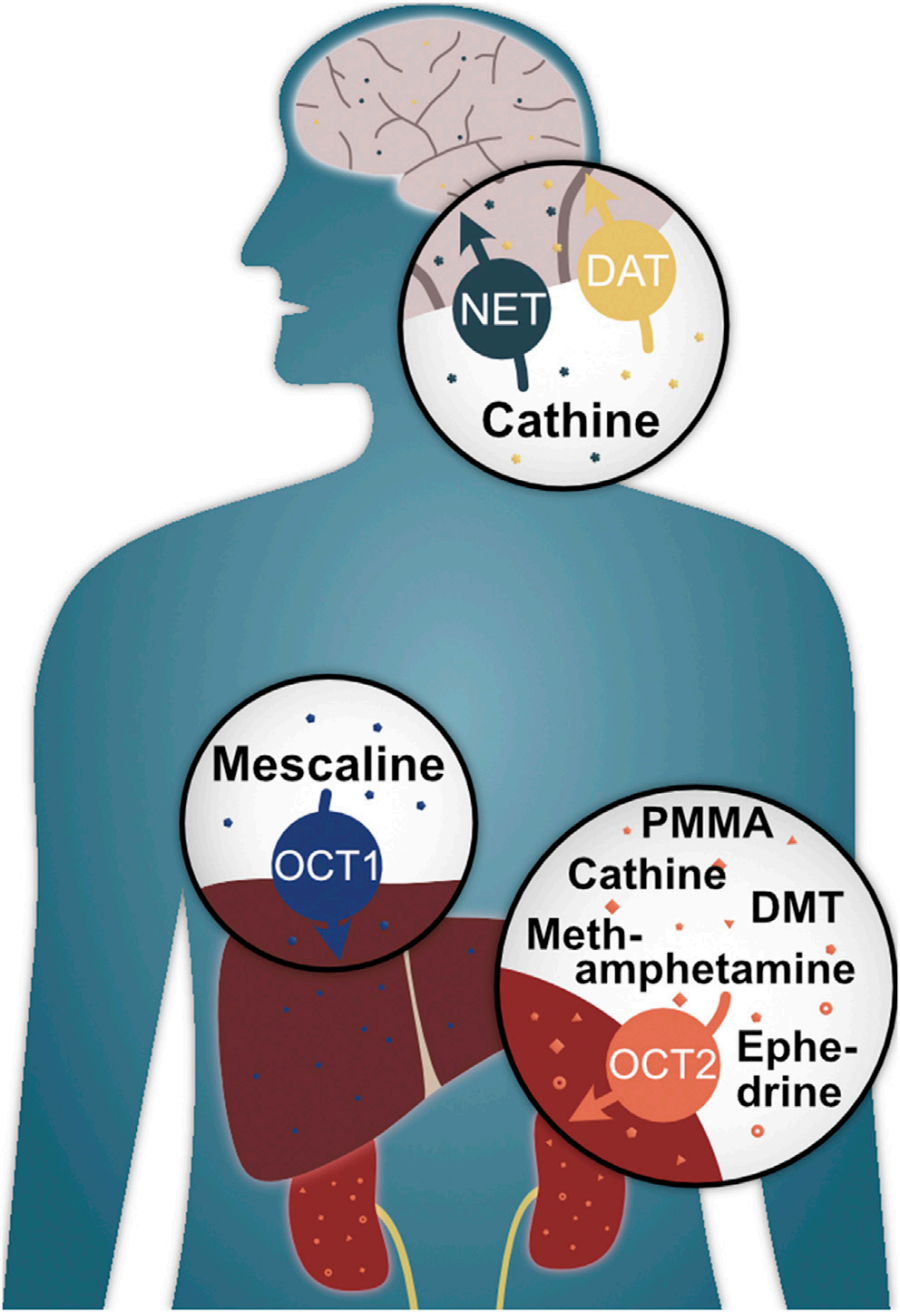
Illustrated summary of key findings of this study and their potential biological relevance.

To summarise, this study has shown that the classic hallucinogen mescaline is a strong substrate of the genetically highly polymorphic OCT1 (Figure 8) and that genetic variants show altered cell uptake, which may have clinical implications. It was also found that the psychoactive compounds methamphetamine, PMMA, (-)-ephedrine, cathine, and DMT are substrates of OCT2 with partially moderate reductions in cell uptake in the A270S variant. Cathine was also discovered to be a substrate of NET and DAT. As to the question of whether OCT1 is a drug trafficker or not, we would argue that it is one indeed. However, it is a very selective one with a clear preference for the hallucinogenic compound mescaline, which is rather unusual for OCT1 given its generally broad substrate profile.

## Data Availability Statement

The raw data supporting the conclusions of this article will be made available by the authors, without undue reservation.

## Author Contributions

Conceptualisation: OJ, MR, and JB; Funding acquisition: MR and JB; Investigation: OJ and LG; Methodology: OJ and JB; Project administration: OJ, MR, and JB; Supervision: JB; Visualisation: OJ and MR; Writing – original draft: OJ and MR; Writing – review and editing: MR and JB.

## Funding

Funded in part by the Deutsche Forschungsgemeinschaft (DFG, German Research Foundation) – Projektnummer 437446827, the research program of the University Medical Center, University of Göttingen, and the Open Access Publication Funds of the University of Göttingen.

## Conflict of Interest

The authors declare that the research was conducted in the absence of any commercial or financial relationships that could be construed as a potential conflict of interest.

## Abbreviations

Abbreviations: CYP, cytochrome P450; DAT, dopamine transporter; DET, diethyltryptamine; DMT, dimethyltryptamine; DOI, 2,5-dimethoxy-4-iodoamphetamine; MATE2-K, multidrug and toxin extrusion protein 2 kidney-specific; MBDB, *N*-methyl-1,3-benzodioxolylbutanamine; MDAI, 5,6-methylenedioxy-2-aminoindane; MDEA, 3,4-methylenedioxy-*N*-ethylamphetamine; MDMA, 3,4-methylenedioxymethamphetamine; MPP^+^, 1-methyl-4-phenylpyridinium; NET, noradrenaline (norepinephrine) transporter; OCT, organic cation transporter; PCR, polymerase chain reaction; PMA, *para*-methoxyamphetamine; PMMA, *para*-methoxymethamphetamine; SERT, serotonin transporter; SLC, solute carrier; WT, wild-type.
