# Supplementary material for: Cellular Uptake of Psychostimulants – Are High- and Low-Affinity Organic Cation Transporters Drug Traffickers?
Source: Front Pharmacol. 2021 Jan 20;11:609811. doi: 10.3389/fphar.2020.609811 (PMC7854383; doi:10.3389/fphar.2020.609811)
Supplement: Supplementary file 1 [file table1.docx]

**Table S1** Mass spectrometry detection parameters of analytes and internal standards

| **Test compound** | **RT^a^ (min)** | **Mass Q1 (Da)** | **Mass Q3 (Da)** | **DP^a^ (V)** | **CE^a^ (V)** | **CXP^a^ (V)** | **Internal standard** |
| --- | --- | --- | --- | --- | --- | --- | --- |
| Amphetamine | 5.8 | 136.0 | 91.0 | 41 | 21 | 16 | ranitidine-d6 |
|  |  |  | (119.0) |  | (13) | (14) |  |
| Methylamphetamine | 6.8 | 150.2 | 91.0 | 31 | 23 | 16 | sumatriptan |
|  |  |  | (119.0) |  | (15) | (8) |  |
| PMA | 3.5 | 166.1 | 149.1 | 36 | 13 | 10 | nadolol |
|  |  |  | (121.1) |  | (25) | (15) |  |
| PMMA | 3.6 | 180.1 | 149.0 | 46 | 17 | 9 | nadolol |
|  |  |  | (121.0) |  | (28) | (22) |  |
| Cathinone | 4.4 | 321.2 | 176.0 | 65 | 25 | 15 | ranitidine-d6 |
|  |  |  | (130.1) |  | (35) | (15) |  |
| Phentermine | 3.6 | 150.2 | 133.0 | 31 | 9 | 8 | nadolol |
|  |  |  | (91.1) |  | (23) | (16) |  |
| (-)-Ephedrine | 4.6 | 166.0 | 148.1 | 41 | 17 | 9 | ranitidine-d6 |
|  |  |  | (133.0) |  | (27) | (8) |  |
| Cathine | 4.3 | 152.1 | 117.0 | 39 | 23 | 22 | ranitidine-d6 |
|  |  |  | (91.0) |  | (39) | (16) |  |
| DOI | 9.7 | 322.0 | 277.0 | 51 | 27 | 18 | guanfacine |
|  |  |  | (302.0) |  | (17) | (20) |  |
| Mescaline | 6.6 | 211.9 | 165.0 | 46 | 31 | 10 | ranitidine-d6 |
|  |  |  | (195.0) |  | (15) | (12) |  |
| MDMA | 3.5 | 193.9 | 163.0 | 41 | 17 | 10 | nadolol |
|  |  |  | (104.9) |  | (33) | (6) |  |
| MDEA | 3.7 | 208.0 | 163.0 | 51 | 19 | 10 | nadolol |
|  |  |  | (105.0) |  | (35) | (20) |  |
| MBDB | 4.0 | 208.0 | 135.0 | 51 | 24 | 8 | nadolol |
|  |  |  | (177.1) |  | (15) | (11) |  |
| MDAI | 5.4 | 178.0 | 161.0 | 43 | 17 | 10 | ranitidine-d6 |
|  |  |  | (131.0) |  | (27) | (16) |  |
| Cocaine | 5.2 | 304.3 | 182.0 | 41 | 27 | 12 | caffeine |
|  |  |  | (77.0) |  | (77) | (14) |  |
| Methylecgonine | 2.9 | 200.2 | 182.1 | 46 | 25 | 12 | metformin |
|  |  |  | (82.0) |  | (35) | (6) |  |
| DMT | 8.0 | 189.2 | 58.1 | 46 | 25 | 10 | fenoterol |
|  |  |  | (143.9) |  | (21) | (8) |  |
| DET | 4.6 | 217.3 | 86.0 | 51 | 19 | 16 | tulobuterol |
|  |  |  | (143.9) |  | (27) | (10) |  |
| Caffeine | 4.5 | 195.2 | 138.1 | 70 | 27 | 8 | - |
|  |  |  | (110.0) |  | (32) | (8) |  |
| Fenoterol | 8.9 | 304.1 | 107.1 | 80 | 44 | 12 | - |
|  |  |  | (135.2) |  | (24) | (12) |  |
| Guanfacine | 6.1 | 246.2 | 59.9 | 36 | 32 | 10 | - |
|  |  |  | 229.2 |  | (9) | (6) |  |
| Metformin | 2.7 | 130.0 | 71.0 | 46 | 35 | 10 | - |
|  |  |  | (60) |  | (19) | (10) |  |
| Nadolol | 3.5 | 310.1 | 254.1 | 66 | 23 | 16 | - |
|  |  |  | 201.0 |  | (31) | (16) |  |
| Ranitidin-d6 | 4.1 | 321.0 | 176.0 | 65 | 25 | 15 | - |
|  |  |  | (130.1) |  | (35) | (15) |  |
| Sumatriptan | 6.3 | 296.2 | 58.2 | 50 | 30 | 12 | - |
|  |  |  | (251.2) |  | (24) | (12) |  |
| Tulobuterol | 4.7 | 228.1 | 153.9 | 60 | 23 | 10 | - |
|  |  |  | (119.1) |  | (41) | (8) |  |

^a^Abbreviations: RT, retention time; DP, declustering potential; CE, collision energy; CXP, collision cell exit potential
