# Supplementary material for: Cellular Uptake of Psychostimulants – Are High- and Low-Affinity Organic Cation Transporters Drug Traffickers?
Source: Front Pharmacol. 2021 Jan 20;11:609811. doi: 10.3389/fphar.2020.609811 (PMC7854383; doi:10.3389/fphar.2020.609811)
Supplement: Supplementary file 2 [file image1.pdf]

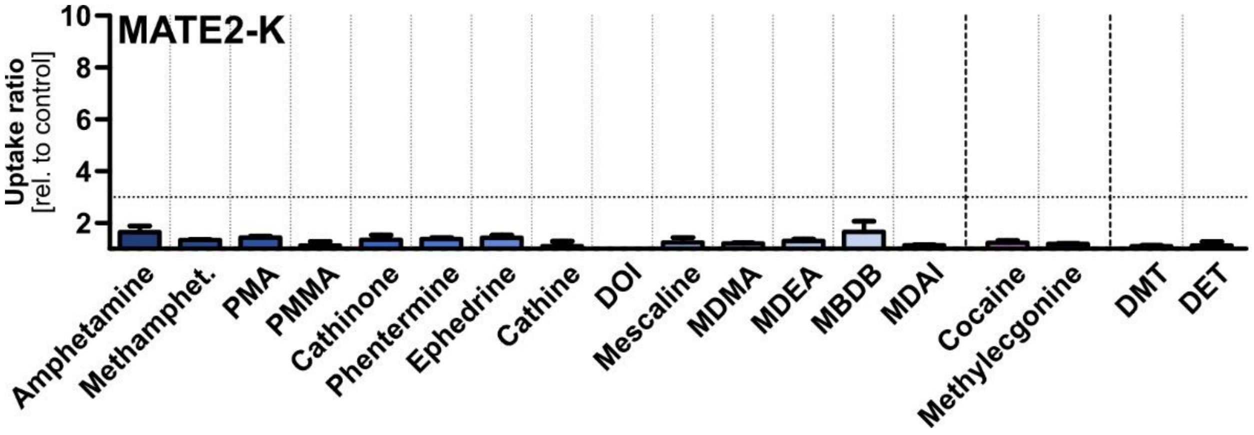

Figure S1 Transport of different psychostimulant and hallucinogenic substances at a concentration of 1 µM by MATE2-K, shown as the ratios of uptake after 1 min in transporter-transfected cells over empty vector control cells. Shown are the mean values of ≥ 3 independent experiments + SEM. The horizontal dotted line indicates an uptake ratio of 3, which was set as the minimum threshold for more detailed characterisation. The uptake in OCT1-overexpressing cells was not found to be significantly different to control cells, according to Student's t-test.
